# Evidence-Based Guidance for One Health Preparedness, Prevention, and Response Strategies to Marburg Virus Disease Outbreaks

**DOI:** 10.3390/diseases12120309

**Published:** 2024-12-02

**Authors:** Claude Mambo Muvunyi, Jean Claude Semuto Ngabonziza, Noella Bigirimana, Nicaise Ndembi, Emmanuel Edwar Siddig, Jean Kaseya, Ayman Ahmed

**Affiliations:** 1 Rwanda Biomedical Center (RBC), Kigali 7162, Rwandaayman.ame.ahmed@gmail.com (A.A.); 2 Department of Clinical Biology, University of Rwanda, Kigali 3900, Rwanda; 3 Research, Innovation and Data Science Division, Rwanda Biomedical Centre, Kigali 7162, Rwanda; 4 The Africa Centres for Disease Control and Prevention (Africa CDC), Ring Road, 16/17, Haile Garment Lafto Square, Addis Ababa P.O. Box 3243, Ethiopia; 5 Unit of Applied Medical Sciences, Faculty of Medical Laboratory Sciences, University of Khartoum, Khartoum 11111, Sudan; 6 Institute of Endemic Diseases, University of Khartoum, Khartoum 11111, Sudan; 7 Pan-Africa One Health Institute (PAOHI), Kigali 11KG ST203, Rwanda

**Keywords:** zoonotic-viral infection, global health security, hemorrhagic fever, multisectoral one health strategy, pandemic-prone diseases, pandemic preparedness, prevention, response, Africa

## Abstract

Objectives: Marburg virus disease (MVD) is on the WHO list for pandemic-prone pathogens. The current outbreak in Rwanda provides an opportunity to map outbreaks and generate information to inform policymaking, resource mobilization, and guide the implementation of cost-effective response strategies. Methods: We synthesized available information about MVD to build holistic, up-to-date evidence to inform policymakers, public health leaders, and healthcare and public health services providers in their development and implementation of cost-effective preparedness, prevention, and control measures. Results: We have identified 20 outbreaks of MVD that occurred in 14 countries between 1967 and 2024; these outbreaks led to 580 confirmed cases and 423 deaths in total. We summarize the available information about the main clinical signs, diagnostic tools, primary reservoir, transmission dynamics, and case management protocol. We also document the best practices in the prevention and control of MVD outbreaks, including the implementation of a multisectoral One Health strategy for preparedness, prevention, and response to MVD outbreaks that incorporates the strict implementation of WASH and infection prevention measures, contact tracing, and the isolation of infected and suspected humans and animals, and enhances the implementation of the International Health Regulations, particularly efficient cross-country coordination. Conclusions: In the absence of a licensed treatment or vaccine for MVD, the response strategy to MVD should focus on preventive measures, including community engagement to promote the reduction in contact between humans and reservoirs, the supportive care and isolation of patients, and proper waste management. High risk populations such as frontline responders, including healthcare providers and community health workers, should be prioritized so that they can access all currently available protection measures.

## 1. Introduction

Marburg virus disease (MVD) is a zoonotic-viral hemorrhagic fever that is transmitted between humans and other animal hosts; this is mainly through fruit bats, which are known as natural reservoirs. It is caused by one of two sibling viruses; the Marburg virus (MARV) and Ravn virus both belong to the species *Orthomarburgvirus marburgense* under the genus *Orthomarburgvirus*, and similar to the Ebola virus, they are all classified within the *Filoviridae* viral family [1]. The disease is characterized by a high fatality rate of up to 90% [2]. The virus was identified for the first time during two simultaneous outbreaks in Germany and Belgrade in Serbia, in 1967 [3]. Due to its zoonotic nature, the disease can be acquired through direct contact with an infected animal and/or human or contaminated products and material [4]. This pathogen poses a significant public health and security threat to human, animal, and environmental health due to its severity, rapid spread, and the absence of a licensed vaccine or treatment [5]. The disease is on the World Health Organization (WHO) and The Global Alliance for Vaccines and Immunizations (GAVI) lists of pathogens that will very likely cause the next pandemic, and the WHO list of high priority diseases for research and development [6,7,8]. The disease is currently causing an outbreak in Rwanda [9].

## 2. Historical Epidemiology

**Figure 1:**
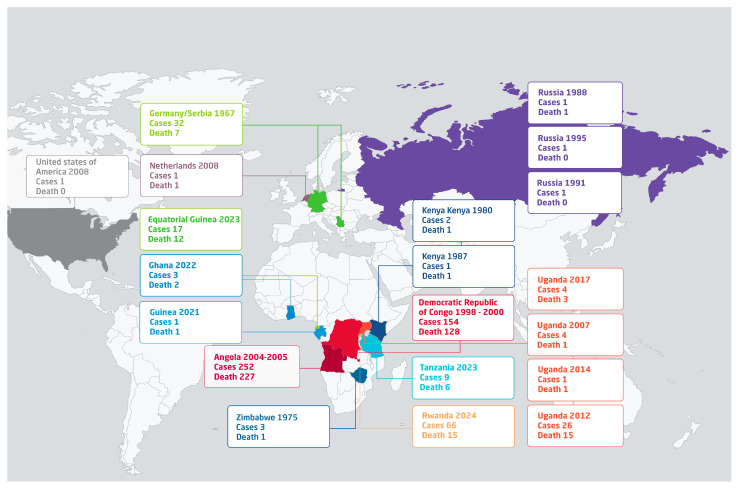
Summary of Marburg virus outbreaks reported worldwide.

The first recorded cases of MVD were among laboratory workers using imported African green monkeys for the production of primary cell cultures [10]. Due to its natural circulation among animal reservoirs that mainly exist in Africa, the disease predominantly affects individuals in Sub-Saharan Africa [11,12]. MARV raises concern because of its high mortality rate and the challenges associated with its management in outbreak situations. To date, there have been 19 reported outbreaks of MARV, in Uganda, Ghana, Guinea, Tanzania, Angola, the Democratic Republic of the Congo (DRC), Zimbabwe, and most recently, Rwanda (Figure 1). Additionally, outbreaks of MARV had occurred in Germany, the Netherlands, Russia, and Serbia in Europe, and in the USA (Figure 1). Most outbreaks of MVD have emerged in Uganda and Russia with four and three outbreaks reported, respectively. It is worth noting that all of the three outbreaks that occurred in Russia between 1988 and 1995 were related to laboratory acquired infection involving a single patient each, which underscores the aggressive pathogenicity and virulence of MARV [13]. However, the largest outbreaks with regard to the number of cases and deaths were reported from Angola (252 cases and 227 deaths) and the DRC (154 cases and 128 deaths) (Figure 1). Overall, over 580 cases and 423 deaths due to MVD were reported worldwide between 1967 and 2024 (Figure 1). The currently ongoing outbreak of MVD in Rwanda has involved 66 cases and 15 deaths, exhibiting around a 23% case fatality rate (Figure 1). 

## 3. Natural Reservoirs and Routes of Transmission

All reported outbreaks of MARV involved contact with animal hosts in nature or laboratory settings, indicating a sustainable epizootic transmission that maintains the virus circulation in the environment. This is supported by recently developed evidence revealing that the currently ongoing outbreak of MVD is sparked by spillover from animal origin [14]. 

Various species of bats have been incriminated in the virus’ transmission and some species have been identified as natural reservoirs for filoviruses including the MVD and Ebola viruses. There is a compelling evidence about the critical role of *Rousettus aegyptiacus*, commonly known as the Egyptian fruit bat, in the ecology and dynamics of MARV [11,15]. Live MARV was successfully isolated from *R. aegyptiacus* bats in Uganda [16,17]. Furthermore, evidence shows that *R. aegyptiacus* bats infected with MARV are asymptomatic, and their flights between endemic and disease-free areas contribute to the cross-country spread of the virus [18,19]. Nonetheless, infected bats have a mild immune response, with the virus being detectable in the liver, spleen, blood, kidney, salivary gland, intestines, and in the inoculated skin site. Viral shedding was detectable through oral and rectal swabs, suggesting the possibility of viral share and transmission due to contamination through oral and anal materials including direct contact, saliva, and excreta as well as vertical transmission [20,21]. Therefore, the inhalation of aerosols or contaminated excreta from these bats, as well as the consumption of infected bats, might be routes of transmission of the virus into the human population. 

In addition to bats and non-human primates, pigs were also incriminated to be an amplifier host for MARV, particularly during the disease outbreaks [22,23]. According to the European Centre for Disease Prevention and Control (ECDC), forest antelopes might be a source of infection with MARV [24]. The previous detection of MARV in partially eaten fruits underscores the environmental risk of the virus and necessitates the need for environmental surveillance to monitor the disease dynamics, particularly in countries within the geographical niche of MARV [12,25].

## 4. Routes of Transmission 

**Figure 2:**
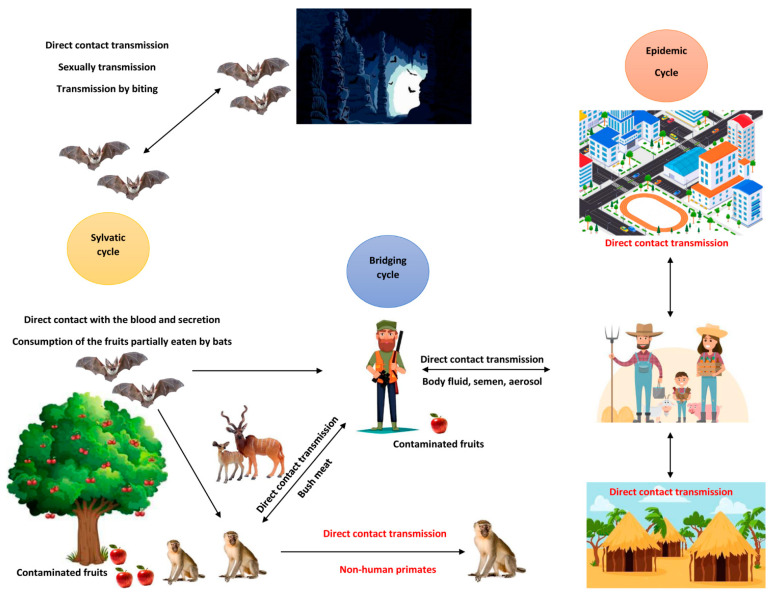
Illustration of the various transmission cycles of Marburg virus in different ecological settings.

MARV transmission occurs primarily through mucosal surfaces, broken skin, or parenteral routes. During outbreaks, direct contact with infected humans or animals is the most common source of infection. Parenteral exposure—often occurring in healthcare settings—is one of the most lethal routes of infection (Figure 2) [26]. 

The 1967 outbreak provides evidence of the human-to-human transmission of MARV [27], wherein the outbreaks resulting from direct contact with the blood and organs of infected monkeys or involvement in post-mortem examinations was shortly followed by a secondary transmission among individuals who had no contact with the originally infected animals or their materials [27]. The human-to-human transmission of MARV typically occurs via direct contact with blood, saliva, sweat, stool, urine, tears, and semen, particularly during the care of infected patients [27,28,29]. Additionally, handling corpses during traditional burial practices is a source of infection [30]. Furthermore, evidence from the 1967 outbreak suggested possible sexual transmission during the convalescence phase, as virus antigens were detected in the semen of an infected patient [27]. Evidence also suggests the mother-to-child transmission of MARV occurring through breastfeeding [29].

Aerosol, droplets, and splashes of liquids from patients are a high risk for the transmission of MARV in a confined space, particularly where ventilation is inadequate [31]. This is of high concern, because it indicates the risk of nosocomial outbreaks of MARV in healthcare facilities, which threatens the lives and health of healthcare providers, community health workers, patients, co-patients, visitors, and supportive staff in these facilities. Furthermore, aerosolized particles can remain suspended in the air for an extended period of time, leading to a rapidly growth in the transmission rate of the disease [32,33]. 

## 5. Clinical Presentations

Marburg virus disease has an incubation period ranging between 2 and 21 days [23]. The incubation period is dependent on factors such as the infectious dose and route of infection. During this incubation period, individuals are not infectious. The transmission occurs after the onset of the disease [5,34]. The clinical course of MVD infection is generally divided into three phases as shown in Table 1. 

The first phase, known as the generalized phase, lasts from days one to four and is characterized by an abrupt onset with nonspecific, flu-like symptoms, including high fever (typically between 39 and 40 °C), severe headache, chills, myalgia, prostration, and malaise [26]. 

The second phase, the early phase, spans from 5 to 13 days, during which 50–75% of patients experience gastrointestinal symptoms, such as anorexia, abdominal discomfort, severe nausea, vomiting, and diarrhea; this is within the first 2 to 5 days of the second phase. The intensity of the disease often escalates between day five and seven, presenting with a maculopapular rash and symptoms of hemorrhagic fever, including petechiae, mucosal and gastrointestinal bleeding, as well as hemorrhages from venipuncture sites [26]. 

The final phase, the convalescence phase, begins after day 13, during which survivors may skip the most severe symptoms and may not reach the late organ phase altogether. Neurological symptoms like disorientation, agitation, seizures, and coma may manifest in this phase [35]. In some cases, complications during the recovery period include joint pain, uveitis, orchitis, and pericarditis, with recovery often being slow. Furthermore, throughout the course of MVD, disseminated intravascular coagulation, lymphopenia, and thrombocytopenia can emerge within a week from the onset of symptoms. Patients ultimately either recover with appropriate supportive care or end up with fatal outcomes, such as dehydration, internal bleeding, and multi-organ failure, within eight to sixteen days after the symptoms begin.

## 6. Diagnosis

MVD diagnosis employs various laboratory-based techniques that are adapted according to the disease’s stages for effective management. Molecular diagnostics, including reverse transcription polymerase chain reaction (RT-PCR), nested RT-PCR, and real-time quantitative RT-PCR (qRT-PCR), which can be performed from blood samples as well as buccal swab, offer the sensitive and specific detection of viral RNA, aiding diagnosis in both the early and late stages [36]. 

In the early stage, the detection of viral antigens in the bloodstream is crucial, utilizing methods such as virus isolation via cell culture, antigen capture enzyme-linked immunosorbent assay (ELISA) with monoclonal antibodies produced with the recombinant nucleoprotein including MAb2A7 and MAb2H6, and the immunohistochemical analysis of tissue samples to identify MARV antigens [37,38]. As the disease progresses, serological assays become essential for detecting IgM and IgG antibodies, employing indirect immunofluorescence assays and IgM/IgG capture ELISA to assess infection history and immune response. 

In addition to the above mentioned specific tests, complementary diagnostic evaluations are essential for physicians who suspect MVD. A Complete Blood Count (CBC) often reveals hallmark laboratory findings such as thrombocytopenia (reduced platelet count) and leukopenia (decreased white blood cell count), both of which are critical indicators of viral infection and the body’s immune response. Moreover, MVD patients frequently exhibit elevated liver enzyme levels, particularly aspartate aminotransferase (AST) and alanine aminotransferase (ALT), which signal hepatic involvement and can provide valuable insight into liver function, aiding in clinical management decisions. Additionally, the presence of proteins in urine samples can indicate renal dysfunction associated with MVD, making urinalysis an important component of a comprehensive patient evaluation. Collectively, these diagnostic tools enhance the clinician’s ability to assess disease severity and tailor interventions effectively. However, these biomarkers are not exclusive to MVD as they can also be associated with arboviral disease infections, so they need to be analyzed cautiously within context to guide the supportive care.

## 7. Case Management

Currently, there are no specific antiviral treatments that are approved and licensed for MVD, so medical management primarily involves supportive care. This includes rehydration, managing symptoms, and maintaining electrolyte balance. 

Experimental treatments, such as Galidesivir (BCX4430), an antiviral agent that functions by terminating RNA chains and inhibiting the activity of viral RNA polymerase, resulted in increased survival rates and reduced levels of viremia in animal studies [39,40]. However, it is important to note that data from human trials regarding Galidesivir’s effectiveness have not yet been published. Nonetheless, initial experimental studies revealed that Galidesivir is safe and tolerable for healthy humans without fatality or severe side effects [41]. 

Promising antiviral therapies also included intravenous Favipiravir (T-705), a broad-spectrum antiviral that resulted in the survival of five out of six *cynomolgus macaques* infected with MARV [42]. In addition, Remdesivir (GS-5734) has shown effectiveness and high protectivity rates against MVD, Ebola, and Sudan virus in non-human primate studies [43,44,45]. It was proven to be safe and tolerable without severe adverse effects, improving the recovery rate, and reducing the severe prognosis of the disease and the mortality rate among humans infected with COVID-19 [46].

For the current outbreak in Rwanda, Remdesivir is being offered experimentally as part of a Post-Exposure Prophylaxis (PEP) regimen, and is also available for separate treatment and in combination with Monoclonal Antibody Treatment (MBP091) [47]. The observed recovery rate is promising; however, further investigations are still underway.

Similarly to the safe burial rituals, it is crucial to engage with traditional healers and community leaders in case management because they can support the improvement of healthcare-seeking behavior and the commitment of individuals to the case management guidelines without conflicting with their religious and traditional beliefs, which could be used to restore trust in the health system. This will reduce hiding cases or seeking treatment outside the health system, both of which, if they happen, would drive community-based transmission and result in the development of large outbreaks or pandemics, depending on the range of people’s activities, dynamics, and movements.

## 8. Integrated Multisectoral One Health Strategy for Prevention and Control of MVD

Investigating the origin and dynamics of the current outbreak of MARV in Rwanda, supported with advanced genomics analysis, revealed the zoonotic origin and association of the virus’ emergence with a spillover from contact with animals [14]. 

Marburg virus disease is a zoonotic-viral pandemic-prone disease and can pose a threat to global health security. Affected countries and countries at risk should invest in strengthening pandemic preparedness, prevention, and response (PPPR) against MVD and other highly fatal and pandemic-prone pathogens [48,49,50]. This should be carried out systematically by frequently implementing national risk assessments and One Health prioritization exercises to identify zoonotic diseases of high priority in each country [51]. The management of MVD should focus on cost-effective prevention and control measures that target the hosts and reservoirs of the virus [22,25,52]. The cost-effectiveness of such a PPPR framework can be enhanced through an integrated multisectoral transdisciplinary One Health strategy that includes integrated collaborative surveillance supported with genomics analysis [53,54,55,56,57,58]. Efforts should be made to strengthen the diagnostic capacity for the early detection of MVD among humans, animals, and the environment; efforts should also be made in tracking and isolating confirmed cases and their contacts to closely monitor them during the standard incubation period of three weeks. Recovered males should avoid non-protected sex until their semen is tested and confirmed to be virus-free [59]. 

Additional public health interventions include raising community awareness about the prevention of infection and improving hygiene and sanitation [60]. Therefore, live communication and the immediate public sharing of information is key for effective community and stakeholders’ engagement [61]. Other major interventions include strengthening the implementation of International Health Regulations such as immediate notification, cross-country coordination, enhancing the diagnosis at points of entry, and monitoring and preventing the cross-border transmission of the virus [60,62,63]. 

Infection prevention and control measures in public facilities including healthcare units, restaurants, airports, and public toilets are important. This also includes enforcing the use of personal protective equipment (PPE) for healthcare providers and community health workers, the strict adherence to hygiene protocols, safe burial practices to prevent post-mortem transmission, and firm medical and personal waste management generated from persons who came in direct and/or indirect contact with confirmed and suspected cases [64]. This highlights the need for transparent and sensitizing engagement of influential people including traditional healers, religious leaders, and community matriarchs to guide and facilitate safe burial with culturally, traditionally, and religiously acceptable rituals to reduce the hiding of cases among the community. Furthermore, in resource limited settings, collaboration with international health organizations can enhance response capabilities by ensuring resources and expertise are mobilized to contain outbreaks effectively. 

Vaccines were proven to be cost-effective interventions in the prevention and response to diseases outbreaks [65,66]; however, Rwanda is currently experimentally vaccinating frontline workers in the response to the ongoing outbreak of MVD, including healthcare providers and community health workers with MARV vaccines. Nonetheless, considering that MARV’s vaccine is still not approved for human use, health authorities should be cautious while administrating the MARV vaccine experimentally for high-risk populations; this sub-population mainly includes frontline responders such as healthcare providers and community health workers [67,68]. Accordingly, these vaccinations should be carried out under close monitoring and observation with immediate availability and accessibility to supportive care whenever it is needed.

Additional One Health preventative interventions include reducing the contact between humans and bats, pigs, and non-human primates [22,23]. Particular attention should be focused on pig farms to prevent direct and indirect contact between pigs and bats and bat excreta-contaminated food to avoid the establishment of zoonotic transmission among livestock. Effective prevention measures of MVD should incorporate the food safety authorities, inspecting pig farms that may have had exposure to bats or their excretion and testing animal products for MARV as well as preventing the selling and consumption of partially eaten fruits and forest products in their routine regulations and practices [12,69]. Careful attention should be given to endangered species, such as the mountain gorilla, as they are susceptible to fatal infection with MARV. Therefore, it important to improve the living environment, such as housing for humans and domestic animals, to avoid bat infestation around human dwellings; furthermore, visiting caves that host bats should be avoided [23]. Avoiding the consumption of food contaminated with bats and/or their excreta and ensuring that animal products are cooked thoroughly are other preventative measures; vaccinating the endangered mountain gorilla is also an option to be considered [23]. Considering the limited available information about the host range of MARV, further studies should consider employing a transdisciplinary One Health approach to investigate the disease ecology, natural reservoirs, and amplifying hosts, and the potential routes of the virus’ transmission [22,25,70].

## 9. Conclusions

Marburg virus is a major threat to global health security and highlights the need for strengthening the pandemic preparedness, prevention, and response (PPPR) framework in countries most at risk of disease emergence and outbreaks through the implementation of an integrated multisectoral and transdisciplinary One Health strategy. Efforts to strengthen PPPR should include improving the diagnostic capacity for its early detection and an integrated collaborative surveillance system that monitors the health metrics and dynamics of zoonotic diseases in humans, animals, and the environment. Other cost-effective public health interventions include health education, effective infection prevention and control, and adequate hygiene and sanitation. Investment in the conduct of research and development of vaccines and treatment for improving the prevention and case management are urgent to prepare, prevent, and respond to future pandemics.

## Figures and Tables

**Table 1:** Summarization of the phases of Marburg virus infection including the duration, characteristics, and symptoms.

Phase	Duration	Characteristics	Symptoms
Generalized Phase	Days 1 to 4	Abrupt onset of nonspecific symptoms	High fever (39–40° C), severe headache, chills, myalgia, prostration, and malaise
Early Phase	Days 5 to 13	50–75% of patients experience gastrointestinal symptoms	Anorexia, abdominal discomfort, severe nausea, vomiting, diarrhea, maculopapular rash, and symptoms of hemorrhagic fever (petechiae, mucosal, and gastrointestinal bleeding, hemorrhage from venipuncture sites)
Convalescence Phase	After Day 13	Survivors may skip the most severe symptoms and may not progress to this phase	Neurological symptoms may present (disorientation, agitation, seizures, and coma), recovery symptoms

## References

[1] Biedenkopf N., Bukreyev A., Chandran K., Di Paola N., Formenty P.B.H., Griffiths A., Hume A.J., Mühlberger E., Netesov S.V., Palacios G. (2024). ICTV Virus Taxonomy Profile: Filoviridae 2024. J. Gen. Virol..

[2] Shifflett K., Marzi A. (2019). Marburg virus pathogenesis – differences and similarities in humans and animal models. Virol. J..

[3] Brauburger K., Hume A.J., Mühlberger E., Olejnik J. (2012). Forty-Five Years of Marburg Virus Research. Viruses.

[4] The World Health Organization (WHO) Marburg Virus Disease. https://www.who.int/health-topics/marburg-virus-disease.

[5] Srivastava S., Sharma D., Kumar S., Sharma A., Rijal R., Asija A., Adhikari S., Rustagi S., Sah S., Al-Qaim Z.H. (2023). Emergence of Marburg virus: A global perspective on fatal outbreaks and clinical challenges. Front. Microbiol..

[6] The Global Alliance for Vaccines and Immunizations (GAVI) The Next Pandemic: Marburg?. https://www.gavi.org/vaccineswork/next-pandemic/marburg.

[7] Prioritizing Diseases for Research and Development in Emergency Contexts. https://www.who.int/activities/prioritizing-diseases-for-research-and-development-in-emergency-contexts.

[8] The World Health Organization (WHO) (2024). Pathogens Prioritization: A Scientific Framework for Epidemic and Pandemic Research Preparedness. https://www.who.int/publications/m/item/pathogens-prioritization-a-scientific-framework-for-epidemic-and-pandemic-research-preparedness.

[9] Rwagasore E., Muvunyi C.M., Butera Y., Nsanzimana S., Condo J. (2024). Rwanda’s seven steps in seven days for managing Marburg virus. Nature.

[10] Ristanović E.S., Kokoškov N.S., Crozier I., Kuhn J.H., Gligić A.S. (2020). A Forgotten Episode of Marburg Virus Disease: Belgrade, Yugoslavia, 1967. Microbiol. Mol. Biol. Rev..

[11] Guito J.C., Prescott J.B., Arnold C.E., Amman B.R., Schuh A.J., Spengler J.R., Sealy T.K., Harmon J.R., Coleman-McCray J.D., Kulcsar K.A. (2020). Asymptomatic Infection of Marburg Virus Reservoir Bats Is Explained by a Strategy of Immunoprotective Disease Tolerance. Curr. Biol..

[12] Amman B.R., Schuh A.J., Albariño C.G., Towner J.S. (2021). Marburg Virus Persistence on Fruit as a Plausible Route of Bat to Primate Filovirus Transmission. Viruses.

[13] Abir M.H., Rahman T., Das A., Etu S.N., Nafiz I.H., Rakib A., Mitra S., Bin Emran T., Dhama K., Islam A. (2022). Pathogenicity and virulence of Marburg virus. Virulence.

[14] Butera Y., Mutesa L., Parker E., Muvunyi R., Umumararungu E., Ayitewala A., Musabyimana J.P., Olono A., Sesonga P., Ogunsanya O. (2024). Genomic Characterization Uncovers Transmission Dynamics of Marburg Virus in Rwanda Following a Single Zoonotic Spillover Event. medRxiv.

[15] Schuh A.J., Amman B.R., Guito J.C., Graziano J.C., Sealy T.K., Kirejczyk S.G.M., Towner J.S. (2022). Natural reservoir Rousettus aegyptiacus bat host model of orthonairovirus infection identifies potential zoonotic spillover mechanisms. Sci. Rep..

[16] Timen A. (2009). Response to Imported Case of Marburg Hemorrhagic Fever, the Netherlands. Emerg. Infect. Dis..

[17] Centers for Disease Control and Prevention Transmission of Colorado Tick Fever Virus by Blood Transfusion—Montana. Morbidity and Mortality Weekly Report 1975. https://stacks.cdc.gov/view/cdc/1031.

[18] Amman B.R., Jones M.E.B., Sealy T.K., Uebelhoer L.S., Schuh A.J., Bird B.H., Coleman-McCray J.D., Martin B.E., Nichol S.T., Towner J.S. (2015). Oral Shedding of Marburg Virus in Experimentally Infected Egyptian Fruit Bats (*Rousettus aegyptiacus*). J. Wildl. Dis..

[19] Paweska J.T., van Vuren P.J., Masumu J., Leman P.A., Grobbelaar A.A., Birkhead M., Clift S., Swanepoel R., Kemp A. (2012). Virological and Serological Findings in Rousettus aegyptiacus Experimentally Inoculated with Vero Cells-Adapted Hogan Strain of Marburg Virus. PLoS ONE.

[20] Paweska J.T., van Vuren P.J., Fenton K.A., Graves K., Grobbelaar A.A., Moolla N., Leman P., Weyer J., Storm N., McCulloch S.D. (2015). Lack of Marburg Virus Transmission from Experimentally Infected to Susceptible In-Contact Egyptian Fruit Bats. J. Infect. Dis..

[21] Amman B.R., Carroll S.A., Reed Z.D., Sealy T.K., Balinandi S., Swanepoel R., Kemp A., Erickson B.R., Comer J.A., Campbell S. (2012). Seasonal Pulses of Marburg Virus Circulation in Juvenile Rousettus aegyptiacus Bats Coincide with Periods of Increased Risk of Human Infection. PLoS Pathog..

[22] Dhama K., Chandran D., Chakraborty S., Yatoo M.I., Islam A., Bhattacharya M., Chakraborty C., Harapan H., Chaicumpa W. (2022). Zoonotic concerns of Marburg virus: Current knowledge and counteracting strategies including One Health approach to limit animal-human interface: An update. Int. J. Surg..

[23] The World Health Organization (WHO) Marburg Virus Disease.. https://www.who.int/news-room/fact-sheets/detail/marburg-virus-disease.

[24] European Centre for Disease Prevention and Control (ECDC) Factsheet for Health Professionals about Marburg Virus Disease.. https://www.ecdc.europa.eu/en/infectious-disease-topics/marburg-virus-disease/factsheet-health-professionals-about-marburg-virus.

[25] Pigott D.M., Golding N., Mylne A., Huang Z., Weiss D.J., Brady O.J., Kraemer M.U.G., Hay S.I. (2015). Mapping the zoonotic niche of Marburg virus disease in Africa. Trans. R. Soc. Trop. Med. Hyg..

[26] Mehedi M., Groseth A., Feldmann H., Ebihara H. (2011). Clinical Aspects of Marburg Hemorrhagic Fever. Futur. Virol..

[27] Martini G.A., Schmidt H.A. (1968). [Spermatogenic transmission of the “Marburg virus”. (Causes of “Marburg simian disease”)]. Klin Wochenschr..

[28] Gear J.S., A Cassel G., Gear A.J., Trappler B., Clausen L., Meyers A.M., Kew M.C., Bothwell T.H., Sher R., Miller G.B. (1975). Outbreake of Marburg virus disease in Johannesburg. BMJ.

[29] Borchert M., Muyembe-Tamfum J.J., Colebunders R., Libande M., Sabue M., Van der Stuyft P. (2002). Short communication: A cluster of Marburg virus disease involving an infant*. Trop. Med. Int. Health.

[30] Bausch D.G., Borchert M., Grein T., Roth C., Swanepoel R., Libande M.L., Talarmin A., Bertherat E., Muyembe-Tamfum J.-J., Tugume B. (2003). Risk Factors for Marburg Hemorrhagic Fever, Democratic Republic of the Congo. Emerg. Infect. Dis..

[31] Alves D.A., Glynn A.R., Steele K.E., Lackemeyer M.G., Garza N.L., Buck J.G., Mech C., Reed D.S. (2010). Aerosol Exposure to the Angola Strain of Marburg Virus Causes Lethal Viral Hemorrhagic Fever in Cynomolgus Macaques. Veter. Pathol..

[32] Geisbert T.W., Daddario-DiCaprio K.M., Geisbert J.B., Reed D.S., Feldmann F., Grolla A., Ströher U., Fritz E.A., Hensley L.E., Jones S.M. (2008). Vesicular stomatitis virus-based vaccines protect nonhuman primates against aerosol challenge with Ebola and Marburg viruses. Vaccine.

[33] Lin K.L., Twenhafel N.A., Connor J.H., Cashman K.A., Shamblin J.D., Donnelly G.C., Esham H.L., Wlazlowski C.B., Johnson J.C., Honko A.N. (2015). Temporal Characterization of Marburg Virus Angola Infection following Aerosol Challenge in Rhesus Macaques. J. Virol..

[34] Mitu R.A., Islam R. (2024). The Current Pathogenicity and Potential Risk Evaluation of Marburg Virus to Cause Mysterious “Disease X”—An Update on Recent Evidences. Environ. Health Insights.

[35] Chauhan L., Matthews E., Piquet A.L., Henao-Martinez A., Franco-Paredes C., Tyler K.L., Beckham D., Pastula D.M. (2022). Nervous System Manifestations of Arboviral Infections. Curr. Trop. Med. Rep..

[36] Kurosaki Y., Grolla A., Fukuma A., Feldmann H., Yasuda J. (2010). Development and Evaluation of a Simple Assay for Marburg Virus Detection Using a Reverse Transcription-Loop-Mediated Isothermal Amplification Method. J. Clin. Microbiol..

[37] Grolla A., Lucht A., Dick D., E Strong J., Feldmann H. (2005). Laboratory diagnosis of Ebola and Marburg hemorrhagic fever. Bull. Soc. Pathol. Exot..

[38] Saijo M., Niikura M., Ikegami T., Kurane I., Kurata T., Morikawa S. (2006). Laboratory Diagnostic Systems for Ebola and Marburg Hemorrhagic Fevers Developed with Recombinant Proteins. Clin. Vaccine Immunol..

[39] Kortepeter M.G., Dierberg K., Shenoy E.S., Cieslak T.J. (2020). Marburg Virus Disease: A Summary for Clinicians. Int. J. Infect. Dis..

[40] Warren T.K., Wells J., Panchal R.G., Stuthman K.S., Garza N.L., Van Tongeren S.A., Dong L., Retterer C.J., Eaton B.P., Pegoraro G. (2014). Protection against filovirus diseases by a novel broad-spectrum nucleoside analogue BCX4430. Nature.

[41] Mathis A., Collins D., Dobo S., Walling D.M., Sheridan W.P., Taylor R. (2022). Pharmacokinetics and Safety of the Nucleoside Analog Antiviral Drug Galidesivir Administered to Healthy Adult Subjects. Clin. Pharmacol. Drug Dev..

[42] Bixler S.L., Bocan T.M., Wells J., Wetzel K.S., Van Tongeren S.A., Dong L., Garza N.L., Donnelly G., Cazares L.H., Nuss J. (2018). Efficacy of favipiravir (T-705) in nonhuman primates infected with Ebola virus or Marburg virus. Antivir. Res..

[43] Porter D.P., Weidner J.M., Gomba L., Bannister R., Blair C., Jordan R., Wells J., Wetzel K., Garza N., Van Tongeren S. (2020). Remdesivir (GS-5734) Is Efficacious in Cynomolgus Macaques Infected with Marburg Virus. J. Infect. Dis..

[44] Cross R.W., Bornholdt Z.A., Prasad A.N., Borisevich V., Agans K.N., Deer D.J., Abelson D.M., Kim D.H., Shestowsky W.S., Campbell L.A. (2021). Combination therapy protects macaques against advanced Marburg virus disease. Nat. Commun..

[45] Cross R.W., Bornholdt Z.A., Prasad A.N., Woolsey C., Borisevich V., Agans K.N., Deer D.J., Abelson D.M., Kim D.H., Shestowsky W.S. (2022). Combination therapy with remdesivir and monoclonal antibodies protects nonhuman primates against advanced Sudan virus disease. J. Clin. Investig..

[46] Bakheit A.H., Darwish H., Darwish I.A., Al-Ghusn A.I. (2023). Chapter Three—Remdesivir. Profiles Drug. Subst. Excip. Relat. Methodol..

[47] Rwanda Ministry of Health Rwanda National Guidelines for Management of Marburg Virus Disease. 2024.

[48] Muvunyi C.M., Bigirimana N., Tuyishime A., Mukagatare I., Ngabonziza J.C., Ahmed A. Initiatives and Strategies to Strengthen the National, Regional, and International Global Health Security: A Case Study of Rwanda Biomedical Centre. https://papers.ssrn.com/sol3/papers.cfm?abstract_id=4957490.

[49] Gashema P., Musafiri T., Ndahimana F., Iradukunda H., Saramba E., Nyakatswau S.T., Gahamanyi N., Iradukunda P.G., Ahmed A., Dzinamarira T. (2024). Mpox in East Africa: Learning from COVID-19 and Ebola to Strengthen Public Health Responses. Viruses.

[50] Bockarie M.J., Hanson J., Ansumana R., Yeboah-Manu D., Zumla A., Lee S.S. (2023). The re-emergence of Marburg virus Disease in West Africa: How prepared is the sub-region for preventing recurrent zoonotic outbreaks?. Int. J. Infect. Dis..

[51] Gashegu M., Ahmed A., Clarisse M., Remera E., Tuyishime A., Rwagasore E., Muhizi D., Kanesa N., Ndayisenga F., Thadee T. (2024). One Health Prioritization for Zoonotic Diseases of Public Health Importance in Rwanda. Lancet.

[52] Ali Y., Siddig E.E., Osman M., Mohamed N.S., Musa A., Ahmed A. (2024). Preparedness, Prevention, Investigation, and Response to the Emergence of Mpox in Khartoum, Sudan in 2022. Preprints.

[53] Remera E., Rwagasore E., Muvunyi C.M., Ahmed A. (2024). Emergence of the first molecularly confirmed outbreak of Rift Valley fever among humans in Rwanda, calls for institutionalizing the One Health strategy. IJID One Health.

[54] Remera E., Rwagasore E., Nsekuye O., Semakula M., Gashegu M., Rutayisire R., Ishema L., Musanabaganwa C., Butera Y., Nsanzimana S. (2024). Rift Valley Fever Epizootic, Rwanda, 2022. Emerg. Infect. Dis..

[55] Ahmed A., Ali Y., Ibrahim N.A., Mohamed S.I., Zinsstag J., Siddig E.E., Mohamed N.S., Muvunyi C.M. (2024). One Health Response for Rift Valley Fever Outbreak in Sudan. Preprints.

[56] Nsengimana I., Juma J., Roesel K., Gasana M.N., Ndayisenga F., Muvunyi C.M., Hakizimana E., Hakizimana J.N., Eastwood G., Chengula A.A. (2024). Genomic Epidemiology of Rift Valley Fever Virus Involved in the 2018 and 2022 Outbreaks in Livestock in Rwanda. Viruses.

[57] Ali Y., Ahmed A., Siddig E.E., Mohamed N.S. (2021). The role of integrated programs in the prevention of COVID-19 in a humanitarian setting. Trans. R. Soc. Trop. Med. Hyg..

[58] Ssemanda J.N., Reij M.W., Bagabe M.C., Muvunyi C.M., Nyamusore J., Joosten H., Zwietering M.H. (2018). Estimates of the burden of illnesses related to foodborne pathogens as from the syndromic surveillance data of 2013 in Rwanda. Microb. Risk Anal..

[59] CDC About Marburg. https://www.cdc.gov/marburg/about/index.html.

[60] Wirsiy F.S., Nkfusai C.N., Bain L.E. (2023). The SPIN Framework to Control and Prevent the Marburg Virus Disease Outbreak in Equatorial Guinea. Pan. Afr. Med. J..

[61] Ahmed A. (2020). Urgent call for a global enforcement of the public sharing of health emergencies data: Lesson learned from serious arboviral disease epidemics in Sudan. Int. Health.

[62] Ahmed A., Mahmoud I., Eldigail M., Elhassan R.M., Weaver S.C. (2021). The Emergence of Rift Valley Fever in Gedaref State Urges the Need for a Cross-Border One Health Strategy and Enforcement of the International Health Regulations. Pathogens.

[63] The World Health Organization (WHO) (2008). International Health Regulations (2005).

[64] Brainard J., Hooper L., Pond K., Edmunds K., Hunter P.R. (2015). Risk factors for transmission of Ebola or Marburg virus disease: A systematic review and meta-analysis. Leuk. Res..

[65] Mohamed N.S., Ali Y., Abdalrahman S., Ahmed A., Siddig E.E. (2022). The use of cholera oral vaccine for containment of the 2019 disease outbreak in Sudan. Trans. R. Soc. Trop. Med. Hyg..

[66] Packham A., Taylor A.E., Karangwa M.-P., Sherry E., Muvunyi C., Green C.A. (2024). Measles Vaccine Coverage and Disease Outbreaks: A Systematic Review of the Early Impact of COVID-19 in Low and Lower-Middle Income Countries. Int. J. Public Health.

[67] Manno D. (2023). Developing a vaccine against Marburg virus disease. Lancet.

[68] O’Donnell K.L., Feldmann F., Kaza B., Clancy C.S., Hanley P.W., Fletcher P., Marzi A. (2023). Rapid protection of nonhuman primates against Marburg virus disease using a single low-dose VSV-based vaccine. EBioMedicine.

[69] Gear J.H.S., Margaretha I. (1988). Handbook of Viral and Rickettsial Hemorrhagic Fevers.

[70] Heeney J.L. (2015). Hidden reservoirs. Nature.

